# CRISPR/Cas9-Mediated Knockout of CGNL1 Confers Resistance to Aflatoxin B1 in Porcine Intestinal Epithelial Cells via Suppressing ROS Generation

**DOI:** 10.3390/ijms27093928

**Published:** 2026-04-28

**Authors:** Yu Yuan, Jianlin Yuan, Die Deng, Jiawen Wu, Xun Zhou, Anan Jiang, Jianmei Wang, Xun Wang, Mingzhou Li, Keren Long, Ling Zhao

**Affiliations:** 1 College of Veterinary Medicine, Sichuan Agricultural University, Chengdu 611130, China; yuyuan0423@stu.sicau.edu.cn (Y.Y.);; 2 State Key Laboratory of Swine and Poultry Breeding Industry, College of Animal Science and Technology, Sichuan Agricultural University, Chengdu 611130, China; jianlinyuan@sicau.edu.cn (J.Y.); dengdie@stu.sicau.edu.cn (D.D.); 2024302123@stu.sicau.edu.cn (J.W.); ajiang@sicau.edu.cn (A.J.); jianmeiwang@sicau.edu.cn (J.W.); xunwang@sicau.edu.cn (X.W.);

**Keywords:** aflatoxin B1, IPEC-J2 cells, anti-inflammatory, antioxidant, transcriptome

## Abstract

Aflatoxin B1 (AFB1) is a prevalent and highly toxic mycotoxin in the food and feed chain and can directly injure the intestinal epithelium. Yet, its upstream determinants linking epithelial stress to cytotoxicity remain insufficiently defined. Here, we used porcine intestinal epithelial IPEC-J2 cells to characterize AFB1-induced cytotoxic and transcriptomic responses and to determine the role of the tight-junction scaffold, Cingulin-like 1 (CGNL1), a candidate gene identified through genome-scale CRISPR knockout library screening. The results showed that AFB1 exposure reduced cell viability in a dose-dependent manner and induced oxidative stress. RNA-seq profiling analysis revealed broad transcriptional remodeling, with activation of inflammatory pathways (including NF-κB and JAK–STAT signaling). Based on our constructed *CGNL1*-knockout IPEC-J2 cell line (*CGNL1*-KO IPEC-J2) using CRISPR/Cas9, it was found that *CGNL1* deficiency markedly alleviated AFB1-induced cytotoxicity and oxidative stress. Comparative transcriptomics analysis showed that *CGNL1* knockout attenuated AFB1-triggered aberrant expression of some *CGNL1*-dependent AFB1-responsive genes related to immune response under AFB1 challenge. Together, these findings identify *CGNL1* as a potential modulator of epithelial susceptibility to AFB1 and support its involvement in the regulation of toxin-induced oxidative response.

## 1. Introduction

AFB1 is a highly toxic and widely encountered mycotoxin in the food and feed chain, posing persistent risks to livestock production and human health [1]. While the liver is classically viewed as a primary target organ, the intestinal epithelium represents the first physiological barrier directly exposed to dietary AFB1. It therefore plays an important role in early toxic responses, barrier failure, and downstream inflammation [2,3]. In vivo and in vitro studies increasingly support the concept that intestinal injury is not merely a secondary consequence of systemic toxicity but a primary event that can reshape mucosal immunity and compromise epithelial homeostasis [3,4,5].

Mechanistically, AFB1 toxicity is strongly influenced by its metabolic activation [6,7]. Cytochrome P450 enzymes convert AFB1 into the highly reactive exo-8,9-epoxide (AFBO), which rapidly forms covalent DNA adducts and initiates genotoxic stress responses [6]. Additionally, AFB1 can induce oxidative stress, mitochondrial dysfunction, and apoptosis, thereby amplifying epithelial injury [8,9]. In porcine intestinal epithelial models, AFB1 exposure has been reported to reduce cell viability, elevate oxidative stress and pro-apoptotic signaling, and disrupt tight junction integrity, leading to altered expression of key junctional proteins and increased epithelial permeability [2,8,10]. Loss of epithelial barrier function is closely coupled with dysregulated mucosal immune signaling [11]. Recent evidence shows that AFB1 exposure can perturb intestinal immune function, affecting antimicrobial defense programs and the balance of innate immune cells [4]. However, the upstream determinants that mediate AFB1-triggered cellular toxicology remain insufficiently understood.

Tight junctions are not only physical seals but also signaling hubs that coordinate cytoskeletal organization [12]. The cingulin family proteins—cingulin (CGN) and its paralog, cingulin-like 1 (CGNL1)—reside in the tight-junction cytoplasmic plaque and regulate Rho-family GTPase signaling, junction assembly, and gene expression [13]. Notably, CGNL1 has been shown to regulate the activity of RAC1 by recruiting Tiam1 and GEF-H1 to epithelial junctions [13,14]. In parallel, CGNL1 can affect Rho signaling [15].

Genome-scale CRISPR knockout library screening is a crucial technique for identifying genes associated with toxin-induced cell death. It has been successfully applied to the screening of toxin receptors, the identification of genes essential for cell death, and the elucidation of the mechanisms underlying the toxic effects of various toxins such as fusaric acid [16], Tc toxins [17], and Clostridium difficile toxin A [18]. In this study, we use the porcine intestinal epithelial IPEC-J2 genome-wide CRISPR knockout library to perform a high-throughput screen to identify the genes that are important to AFB1-induced cell death. We conducted cell viability, ROS accumulation, and antioxidant capacity tests to assess the ability of the impairment of AFB1-induced cytotoxicity in CGNL1-KO IPEC-J2 cells. The results are expected to reveal key signaling nodes mediated by CGNL1, providing new molecular targets for alleviating mycotoxin-induced intestinal damage and enhancing the adaptability of the gut to toxin stress in livestock and potentially humans.

## 2. Results

### 2.1. AFB1-Triggered ROS Accumulation and Impaired Antioxidant Capacity

**Figure 1:**
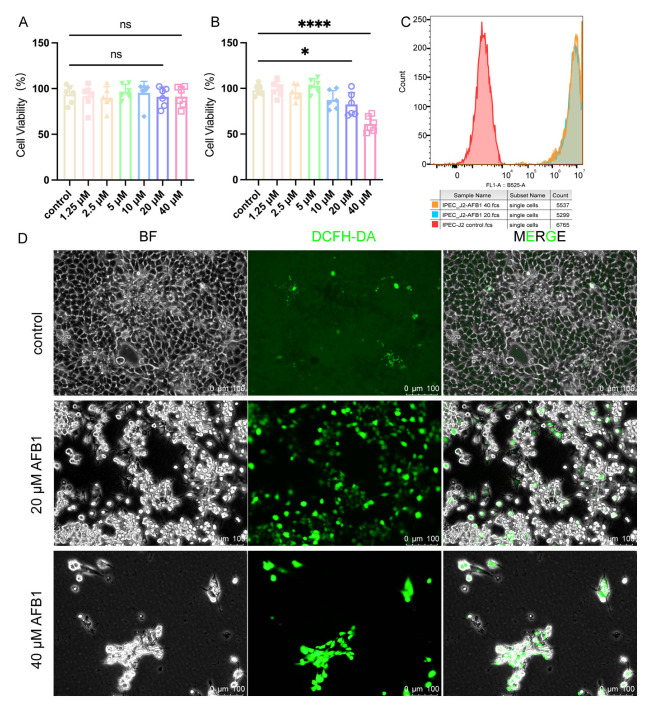
AFB1 induces cytotoxicity and ROS accumulation in IPEC-J2 cells. (**A**) Cell viability of IPEC-J2 cells after 24 h exposure to AFB1 (0, 1.25, 2.5, 5, 10, 20, and 40 μM). (**B**) Cell viability of IPEC-J2 cells after 48 h exposure to AFB1 (0, 1.25, 2.5, 5, 10, 20, and 40 μM). (**C**) Representative flow-cytometry histograms of DCF fluorescence in IPEC-J2 cells after 48 h AFB1 exposure. (**D**) Representative bright-field (BF) DCF fluorescence and merged images showing ROS-associated fluorescence after 48 h AFB1 treatment. Scale bar = 100 μm. Data are presented as mean ± SD, and each symbol represents one replicate. “*” represent *p* < 0.05, “****” represent *p* < 0.0001 vs. control.

After 24 h of exposure, AFB1 did not cause significant cytotoxicity in IPEC-J2 cells across the tested concentration range (Figure 1A). In contrast, after 48 h of treatment, cell viability decreased in a concentration-dependent manner (Figure 1B). No significant cytotoxicity was observed at lower concentrations (1.25–10 μM), whereas significant reductions in viability were detected at 20 μM and became more pronounced at 40 μM. Therefore, 48 h was selected as the exposure duration for subsequent experiments, and 20 μM and 40 μM were chosen as representative toxic concentrations for mechanistic analyses. To further investigate the role of oxidative stress, we used the DCFH-DA probe to detect intracellular ROS levels. Flow cytometry analysis showed a significant rightward shift in DCF fluorescence intensity in the 20 μM and 40 μM AFB1 treatment groups compared to the control group, reflecting increased ROS accumulation (Figure 1C). Fluorescence microscopy consistently showed weak basal fluorescence in the control group cells, while cells exposed to 20 or 40 μM AFB1 exhibited strong green DCF fluorescence (Figure 1D).

**Figure 2:**
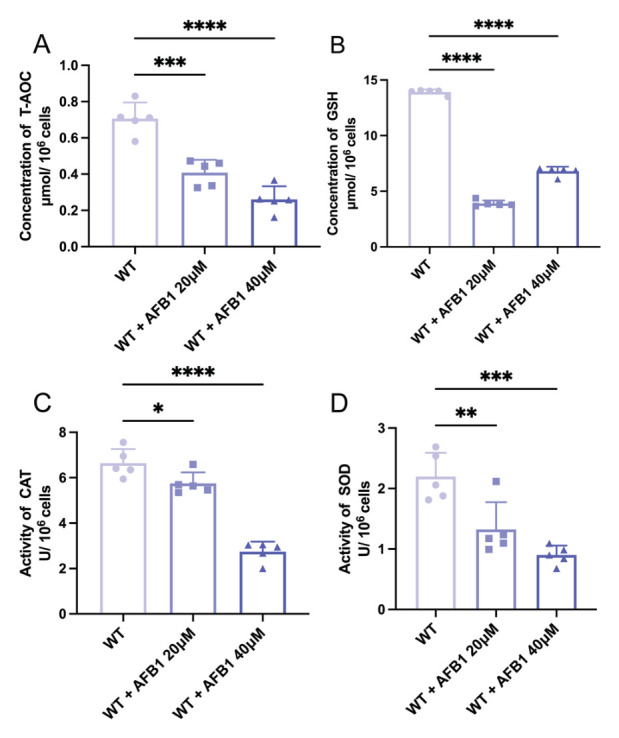
Effects of AFB1 on the antioxidant defense system in IPEC-J2 cells. Cells were treated with 0 μM, 20 μM, or 40 μM AFB1. (**A**) T-AOC. (**B**) GSH concentration. (**C**) CAT activity. (**D**) SOD activity. All antioxidant indices were normalized to 10^6^ cells. Statistical significance was determined compared to the control group: * *p* < 0.05, ** *p* < 0.01, *** *p* < 0.001, **** *p* < 0.0001.

Consistent with the observed ROS overproduction, the endogenous antioxidant defense system of IPEC-J2 cells was severely compromised by AFB1 exposure. We measured the total antioxidant capacity (T-AOC), glutathione (GSH) concentration, and the levels of key antioxidant enzymes (CAT, SOD). As shown in Figure 2, compared with the control group, the T-AOC in cells treated with 20 μM and 40 μM AFB1 exhibited a highly significant, dose-dependent decrease (*p* < 0.001 and *p* < 0.0001, respectively). Meanwhile, the concentration of GSH, a crucial non-enzymatic antioxidant, was sharply reduced following toxin exposure (Figure 2B, *p* < 0.0001). Furthermore, the activities of primary antioxidant enzymes were similarly inhibited. Treatment with 20 μM and 40 μM AFB1 led to significant decreases in catalase (CAT) levels (Figure 2C, *p* < 0.05 and *p* < 0.0001, respectively) and superoxide dismutase (SOD) activity (Figure 2D, *p* < 0.01 for the 20 μM group; *p* < 0.001 for the 40 μM group). Taken together, these indicate that AFB1 induces severe oxidative stress in IPEC-J2 cells not only by promoting ROS generation but also by depleting the cellular antioxidant reserves.

### 2.2. Transcriptomic Profiling of IPEC Cells Following AFB1 Exposure

**Figure 3:**
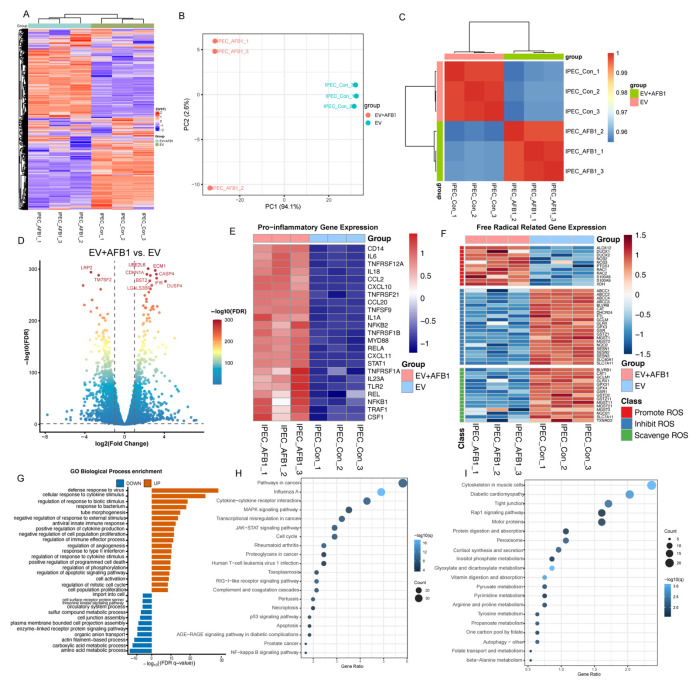
Transcriptomic profiling of EV IPEC-J2 cells following AFB1 exposure. (**A**) Hierarchical clustering heatmap of all genes between control and AFB1-treated groups. (**B**) PCA. (**C**) Sample correlation heatmap based on DESeq2 VST using Pearson correlation. (**D**) Volcano plot of DEGs between AFB1-treated and control groups. Each dot represents one gene. Dots on the right indicate upregulated genes in the EV + AFB1 group, whereas dots on the left indicate downregulated genes. The dashed lines indicate the thresholds for differential expression. The color gradient from blue to red indicates increasing statistical significance. The labeled genes represent representative genes with large expression changes and high significance. (**E**) Inflammation-related DEGs. (**F**) DEGs associated with ROS production and clearance. (**G**) GO enrichment analysis of upregulated and downregulated DEGs. (**H**,**I**) KEGG pathway enrichment analysis of up-regulated (**H**) and down-regulated (**I**) DEGs. EV represents empty-vector IPEC-J2.

To elucidate the effects of AFB1 on the transcriptional level of IPEC-J2 cells, transcriptome sequencing was performed on EV IPEC-J2 cells (IPEC-J2 cells transfected with the empty lentiCRISPR v2 vector) after 48 h of AFB1 exposure. Clustering heatmaps showed clear separation between the AFB1-treated and control groups, with good intra-group biological reproducibility (Figure 3A). Principal component analysis (PCA) revealed significant separation of the transcriptome changes in AFB1-treated cells from those in the control group at PC1 (Figure 3B). Pearson correlation analysis of VST transformation counts showed high intra-group correlation and low inter-group correlation (Figure 3C). In addition, differentially expressed genes (DEGs) identified 1683 genes, with 705 upregulated and 978 downregulated (Figure 3D). AFB1 treatment also significantly altered the expression of multiple inflammation-related genes in IPEC-J2 cells (Figure 3E). Heatmap results showed that the expression profile of free radical-related genes was significantly altered in the AFB1-treated group compared to the IPEC control group. Overall, genes promoting ROS production were upregulated in the AFB1 group, while genes inhibiting ROS production and scavenging ROS were downregulated. (Figure 3F).

To further understand the effects of AFB1 on the transcriptional biological functions of IPEC-J2 cells, we performed GO and KEGG enrichment analyses on upregulated and downregulated DEGs (Figure 3G–I). GO enrichment analysis showed that AFB1 upregulated pathways involved in cell death regulation, such as the regulation of the apoptotic signaling pathway and the positive regulation of programmed cell death. Furthermore, AFB1 significantly downregulated metabolic and transport processes in IPEC-J2 cells, including carboxylic acid metabolism, amino acid metabolism, and organic anion transport activity (Figure 3G). Consistent with GO analysis, KEGG enrichment results showed that AFB1 significantly upregulated signaling pathways in IPEC-J2 cells related to inflammatory stress and apoptosis, such as the NF-κB signaling pathway, JAK–STAT signaling pathway, MAPK signaling pathway, apoptosis, and necrosis (Figure 3H). In addition, pathways related to antioxidants, epithelial structure, and metabolism, such as peroxisomes, pyruvate metabolism, glyoxylate and dicarboxylate metabolism, inositol phosphate metabolism, and tight junctions, were significantly downregulated (Figure 3I). Above all, this indicates that AFB1 induces oxidative stress in IPEC-J2 cells, ultimately leading to cell death.

### 2.3. Genome-Wide CRISPR/Cas9 Screen Nominates CGNL1 as a Candidate Regulator of AFB1-Induced Cytotoxicity

**Figure 4:**
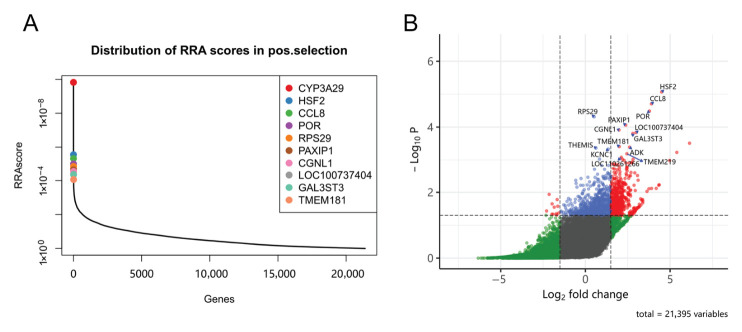
Genome-wide CRISPR/Cas9 identifies candidate genes associated with AFB1-induced cell death. (**A**) Gene-level ranking plot of the positive-selection CRISPR/Cas9 screen in IPEC-J2 cells exposed to AFB1. (**B**) Volcano plot: candidate genes are plotted in a positive screening. Candidate genes were ranked according to sgRNA enrichment in surviving cells relative to the control population.

To identify genes essential for AFB1-induced cell death, we performed a genome-wide CRISPR/Cas9 positive-selection screen in IPEC-J2 cells under AFB1 challenge. Gene-level ranking of enriched sgRNAs identified multiple candidate resistance-associated genes, among which *CGNL1* emerged as an important hit in the positive-selection dataset (Figure 4). The fragments per kilobase of exon model per million mapped fragments value of candidate genes was provided in Table S4.

### 2.4. Validation of CGNL1 Knockout

**Figure 5:**
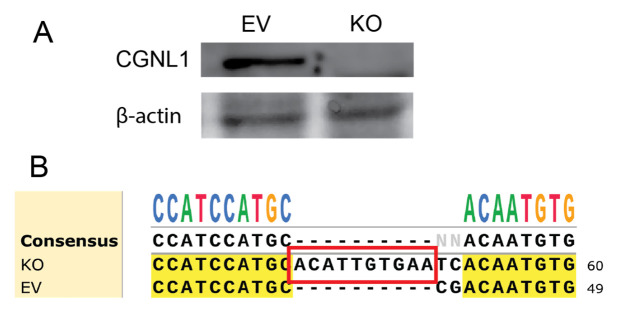
Validation of the CGNL1 knockout generated by targeting exon 1. (**A**) Western blot analysis of CGNL1 expression in EV and CGNL1-KO cells, with β-actin as a loading control. The CGNL1 protein was absent in CGNL1-KO cells compared with EV cells. (**B**) Sanger sequencing of the CRISPR/Cas9-targeted region in exon 1 of CGNL1. The red box marks a 10 bp insertion in exon 1 of CGNL1 detected in CGNL1-KO cells. This insertion is predicted to cause a frameshift mutation, thereby disrupting the CGNL1 coding sequence and resulting in loss of protein function.

Given that *CGNL1* was highlighted by the genome-scale CRISPR/Cas9 screen, we next generated a *CGNL1*-knockout IPEC-J2 cell line to examine its functional contribution to AFB1-induced cytotoxicity. To generate a *CGNL1*-knockout IPEC-J2 cell line, a single-guide RNA (sgRNA) targeting the porcine *CGNL1* locus was designed and cloned into a CRISPR/Cas9 expression vector. The resulting construct contained the U6-driven sgRNA cassette and Cas9 expression module, enabling site-specific genome editing. To validate the CRISPR/Cas9-mediated disruption of *CGNL1*, we examined the edited cells at both the protein and genomic levels. Western blot analysis (Figure 5A) showed that CGNL1 protein was markedly reduced to undetectable levels in the edited cells compared with empty vector (EV) cells. Sanger sequencing (Figure 5B, Supplementary Datas S1 and S2) of the targeted locus identified a 10 bp insertion in exon 1 of *CGNL1* at the CRISPR/Cas9 target site; this insertion is predicted to cause a frameshift. Together, these results confirm the successful generation of *CGNL1*-deficient cells. The predicted protein sequence showed that CGNL1 in EV IPEC-J2 cells consisted of 1294 amino acids, whereas in *CGNL1*-KO IPEC-J2 cells, the insertion of a 10 bp nucleotide sequence caused premature termination of translation, resulting in a truncated CGNL1 protein of only 331 amino acids.

### 2.5. CGNL1 Knockout Attenuates AFB1-Induced Cytotoxicity and Oxidative Stress in IPEC-J2 Cells

**Figure 6:**
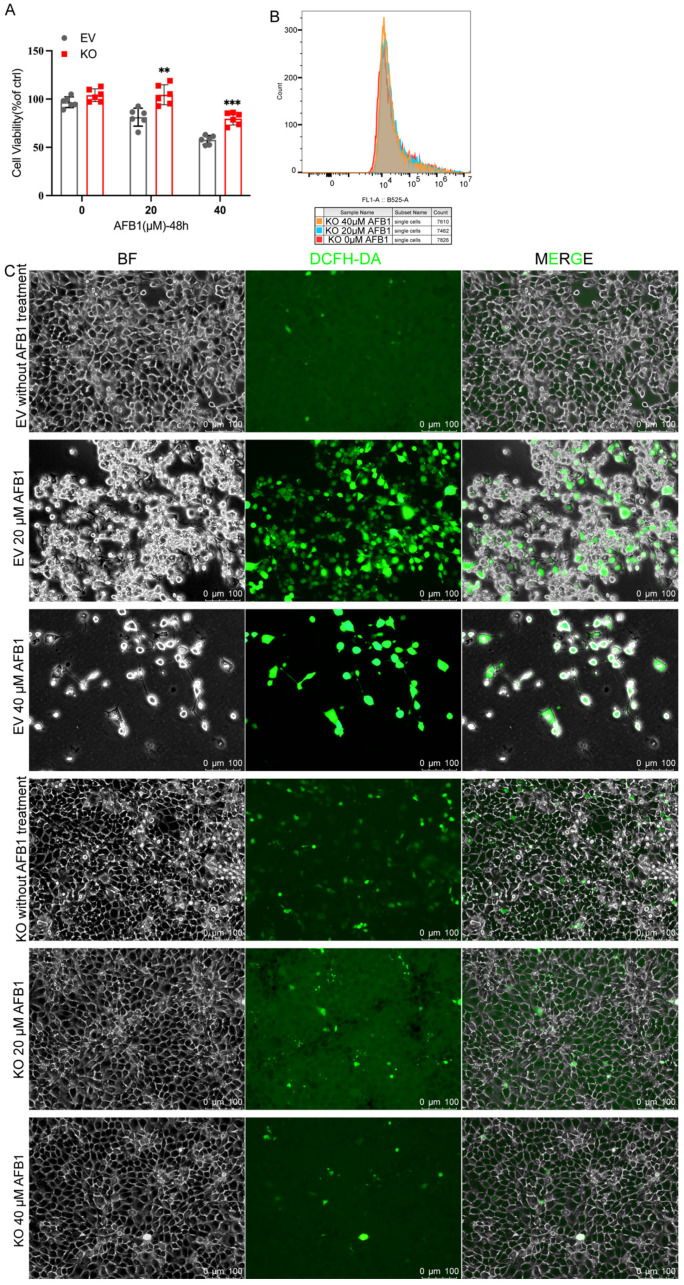
*CGNL1* knockout attenuates AFB1-induced cytotoxicity and ROS accumulation in IPEC-J2 cells. (**A**) Cell viability of EV IPEC-J2 and *CGNL1*-KO IPEC-J2 cells after 48 h exposure to AFB1 (0, 20, and 40 μM). (**B**) Representative flow-cytometry histograms of DCF fluorescence in *CGNL1*-KO IPEC-J2 cells following 48 h AFB1 exposure. (**C**) Representative bright-field (BF), DCF fluorescence, and merged images showing intracellular ROS-associated fluorescence in EV IPEC-J2 and *CGNL1*-KO IPEC-J2 cells after AFB1 treatment. Scale bar = 100 μm. Data are presented as mean ± SD, and each symbol represents one replicate. Statistical significance was determined as indicated (ns, not significant; “**”, *p* < 0.01; “***”, *p* < 0.001). EV represents empty-vector IPEC-J2, and KO represents CGN1-KO IPEC-J2.

To determine the role of *CGNL1* in AFB1-induced epithelial injury, we compared *CGNL1*-knockout IPEC-J2 cells (*CGNL1*-KO IPEC-J2) with EV IPEC-J2 cells (IPEC-J2 cells transfected with the empty lentiCRISPR v2 vector) in cell viability following a 48 h exposure to AFB1 (0, 20, and 40 μM). While baseline viability was similar between the two groups, AFB1 exposure caused a sharp decline in the survival of EV IPEC-J2 cells. In contrast, *CGNL1*-KO IPEC-J2 cells maintained significantly higher viability at both 20 and 40 μM doses (*p* < 0.01 and *p* < 0.001, respectively), demonstrating a strong resistance to AFB1 toxicity (Figure 6A). We assessed intracellular ROS production using a DCFH-DA probe. Flow cytometry revealed only a minor shift in DCF fluorescence in the knockout cells following treatment (Figure 6B). Fluorescence imaging confirmed this observation: while EV IPEC-J2 cells exhibited intense DCF signals after AFB1 challenge, *CGNL1*-KO IPEC-J2 cells showed only faint fluorescence—even under 40 μM AFB1 treatment (Figure 6C).

**Figure 7:**
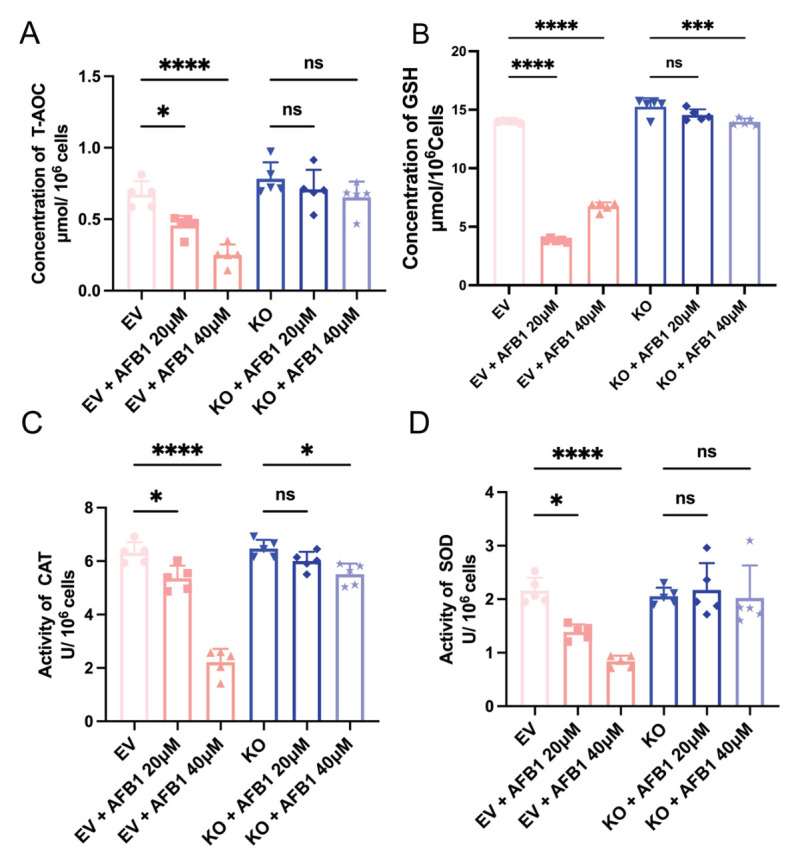
Knockout of *CGNL1* alleviates AFB1-induced impairment of antioxidant defenses in IPEC-J2 cells. EV IPEC-J2 and *CGNL1*-KO IPEC-J2 cells were treated with 0 μM , 20 μM, or 40 μM AFB1 for 48 h. (**A**) T-AOC. (**B**) GSH concentration. (**C**) CAT activity. (**D**) SOD activity. All antioxidant indices were normalized to 10^6^ cells. Statistical significance is indicated as follows: * *p* < 0.05, *** *p* < 0.001, **** *p* < 0.0001; ns, not significant.

Consistent with this marked suppression of ROS accumulation, the deletion of *CGNL1* significantly protected the cellular antioxidant defense system from AFB1-induced impairment. As expected, AFB1 exposure in EV IPEC-J2 cells led to a severe depletion of T-AOC, GSH concentration, and the activities of CAT and SOD (Figure 7). Strikingly, this antioxidant depletion was remarkably alleviated in *CGNL1*-KO IPEC-J2 cells. Notably, even under the severe stress of 40 μM AFB1, the T-AOC concentration and SOD activities in the knockout cells showed no significant difference compared to the untreated knockout control (*p* > 0.05). Furthermore, although 40 μM AFB1 treatment induced a minor decrease in GSH concentration and CAT activity within the knockout group (*p* < 0.001 and *p* < 0.05, respectively), their absolute levels remained substantially higher than those in the corresponding AFB1-treated EV groups, effectively maintaining intracellular redox homeostasis. Taken together, these data indicate that the absence of the *CGNL1* gene confers robust resistance in IPEC-J2 cells against AFB1-induced oxidative damage.

### 2.6. CGNL1 Deficiency Is Associated with Reduced RAC1 Protein Levels in AFB1-Treated IPEC-J2 Cells

**Figure 8:**
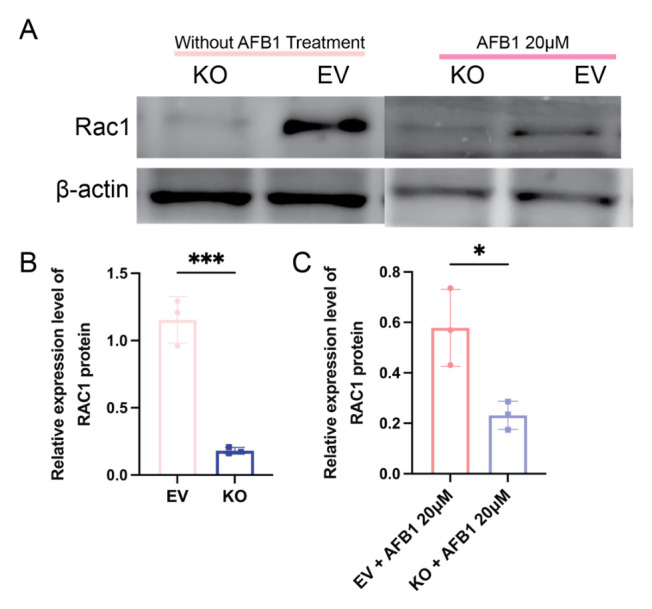
*CGNL1* deficiency is associated with reduced RAC1 protein level. EV IPEC-J2 cells and *CGNL1*-KO IPEC-J2 cells were treated with AFB1 for 48 h. (**A**) Representative Western blot images showing RAC1 protein expression in EV IPEC-J2 and *CGNL1*-KO IPEC-J2 cells under the indicated treatment conditions, with β-actin used as the loading control. (**B**,**C**) Densitometric analysis of RAC1 protein levels normalized to the loading control. Data are presented as mean ± SD from independent experiments. Statistical significance was determined as indicated (* *p* < 0.05, *** *p* < 0.001). EV represents EV IPEC-J2, and KO represents *CGNL1*-KO IPEC-J2.

To further explore the mechanism by which *CGNL1* knockout attenuates AFB1-induced oxidative stress, we examined RAC1 protein expression in EV IPEC-J2 and *CGNL1*-KO IPEC-J2 cells following AFB1 exposure (Figure 8). Western blot analysis showed that the RAC1 protein level was markedly decreased after *CGNL1* knockout. After the exposure to AFB1 for 48 h, the RAC1 protein level remained significantly decreased in the KO cells compared to the EV cells (Figure 8A). Densitometric quantification further confirmed that RAC1 abundance remained significantly lower in the knockout group than in the corresponding EV group under AFB1 challenge (Figure 8B,C). Given the established role of RAC1 in regulating intracellular ROS generation, these results suggest that *CGNL1* deficiency may alleviate AFB1-induced oxidative stress, at least in part, in association with reduced RAC1 protein expression.

### 2.7. CGNL1 Knockout Attenuated Toxin-Induced Transcriptomic Alterations

**Figure 9:**
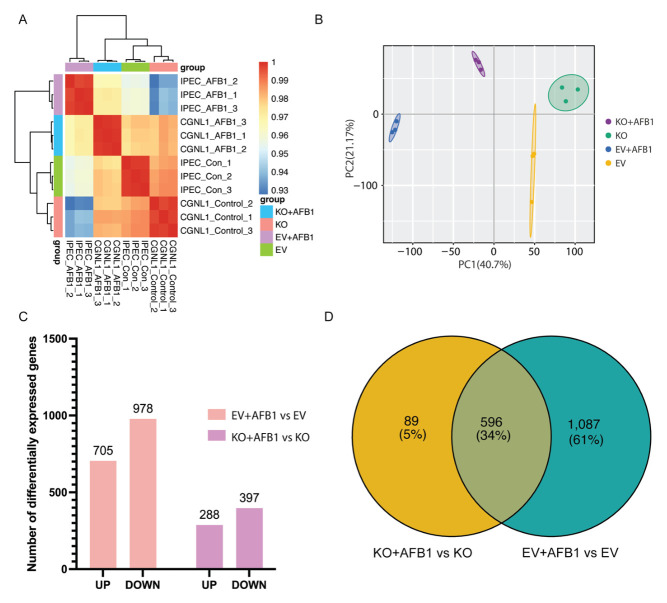
*CGNL1* knockout attenuated toxin-induced transcriptomic alterations. (**A**) Sample-to-sample correlation heatmap. (**B**) PCA of global gene expression profiles. (**C**) Bar plot summarizing the number of upregulated and downregulated DEGs in EV IPEC-J2 + AFB1 vs. EV IPEC-J2 and *CGNL1*-KO IPEC-J2 + AFB1 vs. *CGNL1*-KO IPEC-J2 comparisons. (**D**) Venn diagram showing overlap of DEGs between EV IPEC-J2 + AFB1 vs. EV IPEC-J2 and *CGNL1*-KO IPEC-J2 + AFB1 vs. *CGNL1*-KO IPEC-J2 comparisons. EV represents EV IPEC-J2, and KO represents *CGNL1*-KO IPEC-J2.

To evaluate transcriptomic differences between EV IPEC-J2 and *CGNL1*-KO IPEC-J2 upon AFB1 exposure, we compared global expression patterns and performed differential expression analyses across four groups (EV IPEC-J2, EV IPEC-J2 + AFB1, *CGNL1*-KO IPEC-J2, and *CGNL1*-KO IPEC-J2 + AFB1) (Figure 9A–D). The sample-to-sample correlation heatmap indicated high correlations among biological replicates within each group, supporting good reproducibility (Figure 9A). Notably, the correlation between EV IPEC-J2 + AFB1 and EV IPEC-J2 was relatively lower, whereas *CGNL1*-KO IPEC-J2 + AFB1 remained more similar to *CGNL1*-KO IPEC-J2, suggesting that AFB1-induced transcriptomic perturbations were attenuated in the *CGNL1*-KO IPEC-J2 background and that *CGNL1*-KO IPEC-J2 may partially buffer or reverse AFB1-driven expression changes. PCA also distinguished the four groups in transcriptomic space, with PC1 and PC2 explaining 40.7% and 21.17% of the total variance, respectively, indicating that both CGNL1 knockout (EV IPEC-J2 vs. *CGNL1*-KO IPEC-J2) and AFB1 treatment contribute to the observed transcriptomic variation (Figure 9B). Differential expression statistics revealed a broader transcriptional response to AFB1 in EV IPEC-J2. 705 genes were upregulated and 978 were downregulated in EV IPEC-J2 + AFB1 vs. EV IPEC-J2, whereas 288 genes were upregulated and 397 were downregulated in *CGNL1*-KO IPEC-J2 + AFB1 vs. *CGNL1*-KO IPEC-J2, representing a markedly reduced number of DEGs in *CGNL1*-KO IPEC-J2 (Figure 9C). Venn analysis showed 596 shared DEGs (34%), along with 1087 (61%) specific DEGs in EV IPEC-J2 + AFB1 vs. EV IPEC-J2 and 89 (5%) specific DEGs in *CGNL1*-KO IPEC-J2 + AFB1 vs. *CGNL1*-KO IPEC-J2 (Figure 9D), further supporting that *CGNL1*-KO IPEC-J2 dampens the widespread transcriptional alterations induced by AFB1.

### 2.8. Screening and Functional Analysis of Potential CGNL1-Dependent AFB1-Responsive Genes

**Figure 10:**
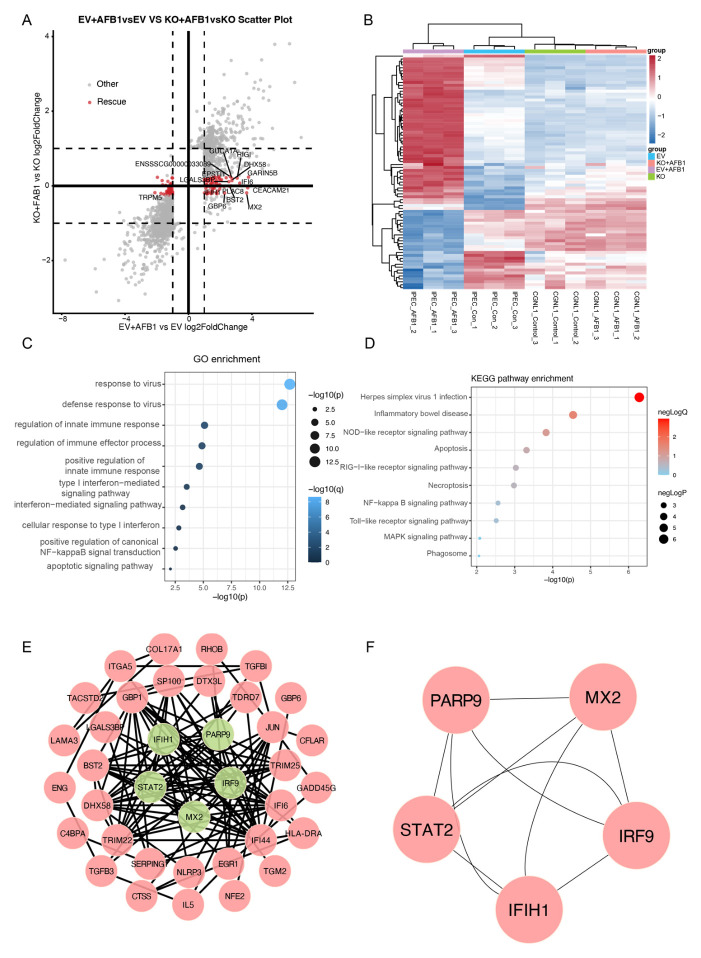
Screening and functional analysis of potential *CGNL1*-dependent AFB1-responsive genes. (**A**) Scatter plot comparing log2 fold changes in EV IPEC-J2+AFB1 vs. EV IPEC-J2 (*x*-axis) and *CGNL1*-KO IPEC-J2+AFB1 vs. *CGNL1*-KO IPEC-J2 (*y*-axis). Genes classified as *CGNL1*-dependent AFB1-responsive genes are highlighted; representative genes are labeled. (**B**) Hierarchical clustering heatmap of 80 *CGNL1*-dependent AFB1-responsive genes. (**C**) GO Biological Process enrichment analysis of the *CGNL1*-dependent AFB1-responsive genes. (**D**) KEGG pathway enrichment analysis of the *CGNL1*-dependent AFB1-responsive genes. (**E**) Protein–protein interaction (PPI) network of *CGNL1*-dependent AFB1-responsive genes. (**F**) PPI network of hub genes. EV represents EV IPEC-J2, KO represents *CGNL1*-KO IPEC-J2.

To identify genes significantly affected by *CGNL1*, we screened for genes where EV was significantly expressed in AFB1 but recovered in KO. We performed a scatter correlation analysis of log2 fold changes between EV + AFB1 vs. EV and KO + AFB1 vs. KO. A total of 80 candidate genes were identified (Figure 10A, Table S3). Hierarchical clustering of 80 candidates showed that, compared with EV IPEC-J2 cells, the overall magnitude of AFB1-induced expression changes was reduced in *CGNL1*-KO IPEC-J2 cells, with expression profiles becoming closer to the untreated state (Figure 10B). GO analysis of the 80 candidate genes revealed significant representation of defense response to virus, response to virus, innate immune response, and cellular response to type I interferon (Figure 10C). At the KEGG analysis, the candidates were significantly enriched in canonical pathways linked to innate immunity, inflammation, and regulated cell death, including the RIG-I-like receptor signaling pathway, NOD-like receptor signaling pathway, and necroptosis (Figure 10D). Through PPI analysis, the network consisted of 37 nodes and 120 edges (Figure 10E). A total of 5 hub genes were identified based on the node score of cytoHubba: *PARP9*, *MX2*, *STAT2*, *IFIH1*, and *IRF9* (Figure 10F).

### 2.9. CGNL1 Knockout Attenuates AFB1-Induced Immune- and Inflammatory-Related Gene Expression in IPEC-J2 Cells

**Figure 11:**
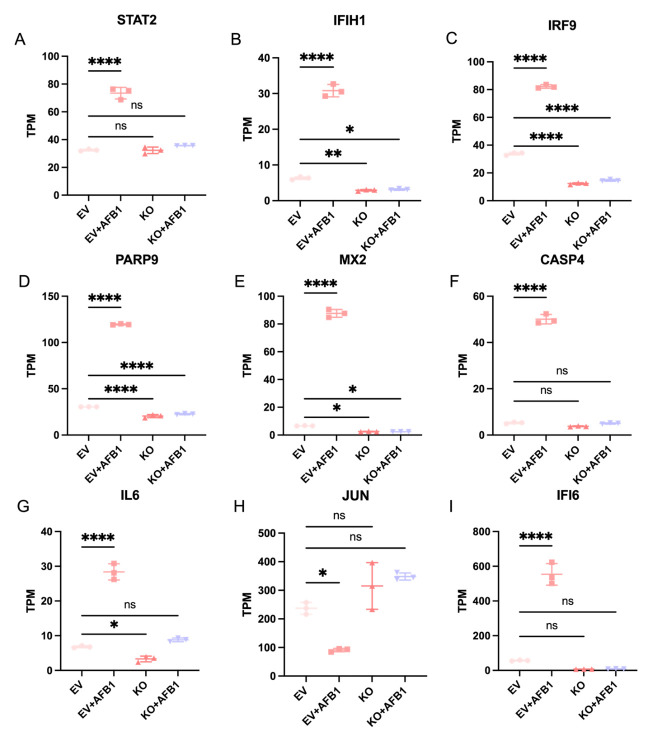
Expression levels of representative differentially expressed genes across experimental groups. The transcript abundance of selected genes was quantified using transcripts per million (TPM) values across different experimental conditions, including EV IPEC cells, *CGNL1*-knockout IPEC-J2 cells, AFB1-treated EV IPEC-J2 cells, and *CGNL1*-KO IPEC-J2 cells treated with AFB1. (**A**–**I**) TPM expression levels of *STAT2* (**A**), *IFIH1* (**B**), *IRF9* (**C**), *PARP9* (**D**), *MX2* (**E**), *CASP4* (**F**), *IL6* (**G**), *JUN* (**H**), and *IFI6* (**I**). Data are presented as individual biological replicates with mean values indicated. Statistical significance was determined as indicated (ns, not significant; “*”, *p* < 0.05; “**”, *p* < 0.01; “****”, *p* < 0.0001). EV represents EV IPEC-J2, and KO represents *CGNL1*-KO IPEC-J2.

Gene expression analysis showed that after AFB1 exposure, multiple genes (including *STAT2*, *IFIH1*, *IRF9*, *MX2*, *PARP9*, *CASP4*, *IL6*, *JUN*, and *IFI6*) were significantly upregulated in the EV IPEC-J2+AFB1 group compared to the EV IPEC-J2 group (Figure 11). In the *CGNL1*-KO IPEC-J2+AFB1 group, the expression levels of these genes returned to a level not significantly different from the *CGNL1*-KO IPEC-J2 group.

### 2.10. qRT-PCR Validation

**Figure 12:**
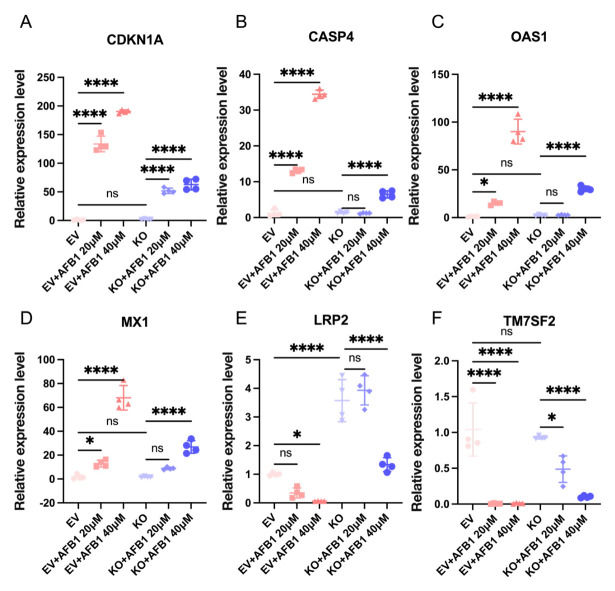
qPCR validation. Relative mRNA expression of (**A**) *CDKN1A*, (**B**) *CASP4*, (**C**) *OAS1*, (**D**) *MX1*, (**E**) *LRP2*, and (**F**) *TM7SF2* in the EV IPEC-J2 and *CGNL1*-KO IPEC-J2 groups after AFB1 (20 or 40 μM) treatment. Groups are shown as EV, EV+AFB1 20 μM, EV + AFB1 40 μM, KO, KO + AFB1 20 μM, and KO + AFB1 40 μM. Data are normalized to a housekeeping gene and presented relative to the corresponding control. Points represent individual replicates; error bars indicate mean ± SD. Statistical significance was determined as indicated (ns, not significant; “*”, *p* < 0.05; “****”, *p* < 0.0001). EV represents EV IPEC-J2, and KO represents *CGNL1*-KO IPEC-J2.

Consistent with the transcriptomic analysis, qRT-PCR validation (Figure 12) showed that AFB1 treatment significantly increased the expression of *CDKN1A*, *CASP4*, *OAS1*, and *MX1* in EV IPEC-J2 cells, and this induction was generally more pronounced at 40 μM than at 20 μM (Figure 12). By contrast, in *CGNL1*-KO IPEC-J2 cells, the AFB1-induced upregulation of these genes was markedly weakened. In addition, *LRP2* and *TM7SF2* were significantly downregulated after AFB1 exposure in EV IPEC-J2 cells, whereas their expression levels were partially restored in *CGNL1*-KO IPEC-J2 cells. Together, these data further confirm that *CGNL1* knockout attenuates AFB1-induced transcriptional perturbations.

## 3. Discussion

AFB1 is widely recognized as a potent foodborne mycotoxin that disrupts intestinal homeostasis, but the molecular events linking epithelial stress to cytotoxicity remain incompletely understood [2,10]. In the present study, we investigated the potential involvement of the tight junction-associated scaffold protein *CGNL1* in the response of porcine intestinal epithelial cells to AFB1. In our AFB1-induced injury model in IPEC-J2 cells, the results further support the view that oxidative stress is an important component of AFB1-induced epithelial damage. Being consistent with previous reports on AFB1-induced epithelial injury [3,8,10], our results showed that AFB1 treatment induced significant oxidative stress. Specifically, AFB1 exposure resulted in impairment of multiple antioxidant-related indices, including GSH, T-AOC, CAT, SOD, and accumulation of ROS, suggesting impaired cellular antioxidant capacity and redox imbalance [8,19]. Transcriptome analysis revealed that AFB1 treatment not only upregulated ROS-related genes and downregulated antioxidant enzymes but also activated classical inflammatory and stress-response pathways (including NF-κB, JAK–STAT, etc.). These findings further confirmed that AFB1 exposure is accompanied by coordinated oxidative and inflammatory-like responses in intestinal epithelial cells, which contribute to the disruption of cellular homeostasis.

Building on our genome-wide CRISPR knockout (GeCKO) screen, *CGNL1* emerged as a candidate regulator of AFB1-induced cytotoxicity. We utilized CRISPR/Cas9 to genetically ablate *CGNL1* in IPEC-J2 cells and investigated how this tight junction-associated scaffold protein modulates the porcine intestinal epithelial response to AFB1-induced cytotoxicity. Intriguingly, we observed that the knockout of *CGNL1* significantly inhibited ROS accumulation, preserved antioxidant capacity, and improved the survival at lethal doses of AFB1, suggesting that *CGNL1* is not merely a passive structural scaffold but likely functions as a pivotal pro-toxic signaling node. Mechanistically, transcriptomic analysis revealed that *CGNL1* deficiency significantly attenuated AFB1-induced transcriptional perturbations. Specifically, while AFB1 exposure elicited a robust response in EV cells with 1683 DEGs, this response was markedly dampened in *CGNL1*-KO cells, where the number of DEGs plummeted to 685. This differential response underscores the critical role of *CGNL1*-mediated signaling in AFB1-induced toxicity. Previous studies have shown that *CGNL1* is a tight-junction-associated scaffold protein involved in the regulation of Rac1 and RhoA signaling [14,20]. Once the activity of these small GTPases changes, their downstream signal pathway can be activated to regulate multiple aspects of cell behavior [21]. In the present study, we observed that RAC1 protein abundance was lower in *CGNL1*-KO cells than in EV control cells under AFB1 treatment. Given the established role of RAC1-related signaling in ROS generation and stress responses, this finding is consistent with the possibility that *CGNL1* deficiency attenuates AFB1-induced oxidative stress at least partly through a Rac1-associated pathway. However, our current data do not directly demonstrate changes in Rac1 activity, nor do they establish a definitive causal signaling cascade downstream of *CGNL1*. Therefore, the mechanistic interpretation should be considered supportive rather than conclusive, and future studies will be required to directly evaluate Rac1 activity and related downstream signaling events.

To further elucidate the transcriptional programs potentially associated with this phenotype, we next focused on the identification of *CGNL1*-dependent AFB1-responsive genes that were markedly dysregulated by AFB1 in EV IPEC-J2 cells but partially normalized in *CGNL1*-KO IPEC-J2 cells. GO and KEGG enrichment analyses indicated that these *CGNL1*-dependent AFB1-responsive genes were highly enriched in immune response-related terms and pathways, including defense response to virus, regulation of innate immune response, type I interferon-mediated signaling pathway, necroptosis, etc. Among these genes, *IFIH1*, a typical cytoplasmic pattern recognition receptor (PRR), functions as a sentinel for endogenous nucleic acid leakage, potentially triggering downstream type I interferon [22,23]. *STAT2* and *IRF9* are key transcriptional mediators of interferon-triggered JAK-STAT signaling and play important roles in antiviral responses and interferon-stimulated gene expression [24,25]. Simultaneously, *MX2*, a typical interferon-induced effector molecule, was significantly up-regulated and may participate in host defense, particularly antiviral defense [26,27]. *PARP9* has been reported to enhance interferon-dependent immune responses by promoting IFN signal transduction [28]. From reported studies, the aberrant expression of these genes is frequently induced or activated in the context of viral infection and interferon responses [26,28,29,30]. In the context of the present study, these findings do not necessarily indicate a canonical antiviral response but rather suggest that AFB1 exposure induces a broader stress-associated inflammatory transcriptional state in intestinal epithelial cells and that this response is attenuated when *CGNL1* is absent. Thus, *CGNL1* may participate in amplifying epithelial stress signaling triggered by AFB1.

Overall, *CGNL1* deficiency is associated with increased tolerance to AFB1-induced injury in IPEC-J2 cells, accompanied by reduced ROS accumulation, preservation of intracellular redox balance, and attenuation of inflammatory/stress-related transcriptional remodeling. Our results demonstrated that *CGNL1* is a potential modulator of epithelial responses to AFB1. At the same time, limitations of the present study should be acknowledged. β-actin was used as the internal reference gene for qRT-PCR validation. The use of a single housekeeping gene in qRT-PCR may represent a limitation. Future studies should systematically assess the stability of candidate reference genes and adopt multiple internal controls for more robust normalization. In addition, all mechanistic analyses were performed in the IPEC-J2 cell line, without additional validation in primary intestinal epithelial cells. While IPEC-J2 is a well-established porcine intestinal epithelial model and is widely used for mechanistic investigations of epithelial injury and toxic responses, the absence of primary-cell validation may limit the translational relevance of the current findings. Therefore, our conclusions should be interpreted within the context of an in vitro mechanistic study. Future work using primary cells and in vivo approaches will be important to further verify the role of *CGNL1* in AFB1-induced intestinal epithelial injury.

## 4. Materials and Methods

### 4.1. Materials and Reagents

All chemical reagents and assay kits used in this study were purchased from commercial suppliers. AFB1 was obtained from Qingdao Pribolab Co., Ltd. (Qingdao, China) and dissolved in dimethyl sulfoxide (DMSO; Merck, Darmstadt, Germany) to prepare a 50 mM stock solution. Cell Counting Kit-8 (CCK-8) was purchased from Dalian Meilun Biotech Co., Ltd. (Dalian, China). The glutathione (GSH) assay kit, Superoxide Dismutase (SOD) assay kit, and Catalase (CAT) assay kit were obtained from Nanjing Jiancheng Bioengineering Institute (Nanjing, China). The total antioxidant capacity (T-AOC) assay kit was obtained from Beijing Boxbio Science and Technology Co., Ltd. (Beijing, China). The DCFH-DA probe was purchased from Solarbio (Beijing, China). TRIzol reagent was obtained from Thermo Fisher Scientific (Waltham, MA, USA). All-in-One cDNA Synthesis SuperMix and SYBR qPCR SuperMix Plus were purchased from NovoProtein (Shanghai, China). Dulbecco’s modified Eagle’s medium (DMEM, high glucose) was obtained from Gibco (Grand Island, NY, USA). Fetal bovine serum (FBS) was purchased from Zhejiang Tianhang Biotechnology (Hangzhou, China). The lentiCRISPR v2 vector (Addgene plasmid #52961), psPAX2 (Addgene plasmid #12260), and pMD2.G (Addgene plasmid #12259) were obtained from Addgene (Watertown, MA, USA). Lipofectamine 3000 was purchased from Invitrogen (Carlsbad, CA, USA). Anti-CGNL1 antibody (Cat. No. FNab01626) was obtained from Wuhan Fine Biotech Co., Ltd. (Wuhan, China). Anti-RAC1 antibody (Cat. No. 66122-1-Ig) and anti-β-Actin antibody (Cat. No. 66009-1-Ig) were supplied by Proteintech Group, Inc. (Wuhan, China).

### 4.2. Cell Culture

The porcine intestinal epithelial cell line IPEC-J2 was obtained from the Beijing North Biological Technology Research Institute (BNCC338128, Beijing, China). Cells were cultured in DMEM supplemented with 10% FBS and 100 U/mL penicillin–streptomycin at 37 °C in a humidified incubator containing 5% CO_2_. Cells were routinely passaged at 70–90% confluence using 0.25% trypsin–EDTA. *CGNL1*-knockout IPEC-J2 (CGNL1-KO IPEC-J2) cells and IPEC-J2 cells transduced with the empty lentiCRISPR v2 vector (expressing Cas9 but lacking a targeting sgRNA, designated as EV, EV IPEC-J2) were cultured under the same conditions.

### 4.3. Genome-Scale CRISPR/Cas9 Knockout Screen Under AFB1 Selection

To identify genes associated with AFB1-induced cytotoxicity, the IPEC-J2 genome-wide knockout library preserved in our laboratory was subjected to screening. A total of three rounds of selection were performed with increasing concentrations of AFB1 (40, 60, and 80 μM). After completion of the three screening rounds, the surviving cells were collected, and genomic DNA was extracted from both the selected cell population and the control library for high-throughput sequencing. Data was analyzed by MAGeCK.

### 4.4. Construction of CGNL1 Knockout IPEC-J2 Cells

*CGNL1*-knockout IPEC-J2 (*CGNL1*-KO IPEC-J2) cells were generated using a lentiviral CRISPR/Cas9 system. Single-guide RNAs (sgRNAs) targeting porcine *CGNL1* were designed using the CHOPCHOP online tool (https://chopchop.cbu.uib.no, accessed on 17 April 2026) and cloned into the lentiCRISPR v2 vector. The sgRNA sequences are listed in Table S1. HEK293T cells were co-transfected with the constructed lentiCRISPR v2-*CGNL1*-sgRNA plasmid, psPAX2, and pMD2.G using Lipofectamine 3000 (Thermo Fisher Scientific, Waltham, MA, USA) at a plasmid ratio of 5:3:2. Viral supernatants were collected at 48 and 72 h post-transfection, filtered through a 0.45 μm membrane filter, and stored at −80 °C. When IPEC-J2 cells reached approximately 50% confluence, they were infected with lentivirus in the presence of polybrene (7 μg/mL; Solarbio, Beijing, China) for 48 h. After infection, cells were selected with puromycin (7 μg/mL; Solarbio, Beijing, China) for 10 days to establish a stable *CGNL1*-knockout cell line. Single-cell clones were isolated by limiting dilution and expanded in culture. Monoclonal cells were collected for genomic DNA extraction. Genomic DNA was extracted using a TIANGEN genomic DNA extraction kit (TIANGEN, Beijing, China) according to the manufacturer’s instructions.

### 4.5. Cell Viability Assay

To determine an appropriate exposure duration and concentration range for subsequent mechanistic experiments, IPEC-J2 cells were initially treated with a gradient of AFB1 concentrations (0, 1.25, 2.5, 5, 10, 20, and 40 μM) for 24 h and 48 h, with DMSO used as the vehicle control. To assess the role of *CGNL1* in AFB1-induced cytotoxicity, EV IPEC-J2 cells and *CGNL1*-KO IPEC-J2 cells were treated with 0, 20, or 40 μM AFB1 for 48 h. Each group contained six replicate wells for cell viability analysis. After treatment, the culture medium was removed. The cells were washed twice with PBS, and 100 μL of DMEM containing 10% CCK-8 solution was added to each well. The plate was then incubated in the dark at 37 °C for 2 h. Absorbance at 450 nm was measured using a microplate reader. Cell viability was calculated as:

$$cell\;viability\;(\%)=\frac{\left(A1-A0\right)}{\left(A2-A0\right)}\times 100\%$$

where A1 represents the absorbance of the AFB1 treatment group, while A0 represents the absorbance of the blank control group, and A2 is the absorbance of the vehicle group.

### 4.6. ROS Assessment

Intracellular ROS levels were measured using the DCFH-DA fluorescent probe. WT IPEC-J2 cells, *CGNL1*-KO IPEC-J2 cells, and EV IPEC-J2 cells were assigned to three groups and exposed to 0, 20, or 40 μM AFB1 for 48 h. After treatment, the culture medium was removed, and the cells were washed twice with PBS. The cells were then incubated with 10 μM DCFH-DA diluted in DMEM at 37 °C for 20 min in the dark. After incubation, the cells were washed three times with DMEM to remove excess probe. ROS fluorescence was observed using a fluorescence microscope (BX63, Olympus, Tokyo, Japan) and quantified by flow cytometry (CytoFLEX, Beckman, Brea, CA, USA).

### 4.7. GSH Assay

Intracellular GSH levels were measured using a commercial assay kit. WT IPEC-J2 cells, *CGNL1*-KO IPEC-J2 cells, and EV IPEC-J2 cells were treated with 0, 20, or 40 μM AFB1 for 48 h. After treatment, the cells were collected and lysed on ice. Following centrifugation at 3500× *g* for 10 min at 4 °C, the supernatants were collected for biochemical analysis according to the manufacturer’s instructions. GSH levels were normalized to cell number and expressed as μmol/10^6^ cells.

### 4.8. SOD Assay

To evaluate the effect of AFB1 on intracellular SOD activity in cells, the cells were grouped and treated as described above. After treatment, the culture medium was discarded, and the cells were gently washed twice with pre-cooled PBS to remove residual medium and any incompletely reacted drug components. The cells were then collected for subsequent analysis. According to the instructions of the SOD assay kit, lysis buffer was added to the collected cells, followed by lysis in an ice bath and centrifugation. The supernatant was collected as the sample for analysis. Blank wells, control wells, and sample wells were prepared according to the kit instructions, and the corresponding detection reagents were added to each well. After thorough mixing, the reaction mixtures were incubated at the specified temperature for an appropriate period to allow full reaction. At the end of the reaction, the absorbance of each well was measured at the appropriate wavelength using a microplate reader. SOD activity was normalized to cell number and expressed as U/10^6^ cells.

### 4.9. T-AOC Assay

To evaluate the effect of AFB1 on T-AOC content in cells, the cells were grouped and treated as described above. After treatment, the culture medium was discarded, and the cells were gently washed twice with pre-cooled PBS to remove residual medium and any incompletely reacted drug components. The cells were then collected for subsequent analysis. Cell lysis was performed according to the instructions of the T-AOC assay kit, followed by centrifugation, and the supernatant was collected as the sample for analysis. Blank wells and sample wells were prepared according to the kit instructions, and the corresponding detection reagents were added to each well. After thorough mixing, the reaction mixtures were allowed to react under the specified conditions, enabling the samples to react with the relevant reagents to form a colored product. At the end of the reaction, the absorbance of each well was measured using a microplate reader. T-AOC levels were normalized to cell number and expressed as μmol/10^6^ cells.

### 4.10. CAT Assay

To evaluate the effect of AFB1 on intracellular CAT activity in WT IPEC-J2 cells, EV IPEC-J2 cells, and *CGNL1*-KO IPEC-J2 cells, the cells were grouped and treated as described above. After treatment, CAT activity was determined according to the instructions provided with the commercial assay kit. Briefly, cells were collected and counted, followed by cell lysis. The lysates were then centrifuged at 2000 rpm for 10 min, and the supernatants were collected for CAT measurement. According to the kit instructions, control tubes and assay tubes were prepared separately, and the corresponding reagents were added to each tube. After thorough mixing, the samples were incubated under the specified conditions. At the end of the reaction, the absorbance was measured at 405 nm. CAT activity was normalized to cell number and expressed as U/10^6^ cells.

### 4.11. Western Blotting

After treatment, the culture medium was discarded, and the cells were gently washed twice with pre-cooled PBS. Total cellular proteins were extracted using RIPA lysis buffer containing protease and phosphatase inhibitors. The lysates were incubated on ice for 30 min and then centrifuged at 12,000× *g* for 15 min at 4 °C. The supernatants were collected.

Proteins were mixed with loading buffer, denatured by boiling at 95 °C for 5 min, and separated by SDS-PAGE. The resolved proteins were then transferred onto PVDF membranes. After transfer, the membranes were blocked with 5% bovine serum albumin (BSA) in TBST for 1 h at room temperature and then incubated overnight at 4 °C with the corresponding primary antibodies. After washing five times with TBST, the membranes were incubated with HRP-conjugated secondary antibodies for 1 h at room temperature. Protein bands were visualized using an enhanced chemiluminescence (ECL) detection reagent and captured using a chemiluminescence imaging system. Band intensities were quantified using ImageJ software (version 1.54), and the expression levels of target proteins were normalized to those of β-actin.

### 4.12. RNA Extraction and Quantitative Real-Time PCR

Quantitative real-time PCR (qRT-PCR) was performed to determine the mRNA expression levels of selected genes. *CGNL1*-KO IPEC-J2 cells and EV IPEC-J2 cells were assigned to three groups: 0, 20, or 40 μM AFB1 for 48 h. After treatment, cells were collected, and total RNA was extracted using TRIzol reagent according to the manufacturer’s instructions. RNA concentration and purity were assessed using a NanoDrop 2000 spectrophotometer (Thermo Fisher Scientific, Wilmington, DE, USA). cDNA was synthesized using NovoScript Plus All-in-one cDNA Synthesis SuperMix (Novoprotein, Shanghai, China). qRT-PCR was performed on a LightCycler 480 II Real-Time PCR System (Roche Diagnostics, Basel, Switzerland). Primers used in this research are listed in Table S2. The thermal cycling conditions were as follows: initial denaturation at 95 °C for 3 min; 40 cycles of denaturation at 95 °C for 10 s and annealing/extension at 60 °C for 30 s. The mRNA expression levels of *CDKN1A, CASP4*, *OAS1*, *MX1*, *LRP2*, and *TM7SF2* were analyzed by the 2^−ΔΔCt^ method, with *β-actin* used as the internal reference gene.

### 4.13. Transcriptome Sequencing and Bioinformatic Analysis

RNA-seq profiling was performed to assess the effects of AFB1 exposure and CGNL1 knockout on gene expression in IPEC-J2 cells. EV IPEC-J2 cells and CGNL1-KO IPEC-J2 cells were each assigned to two treatment conditions, with or without AFB1 exposure for 48 h. Consequently, four groups were included for transcriptome analysis: EV IPEC-J2 cells, EV IPEC-J2 cells + AFB1, CGNL1-KO IPEC-J2, and CGNL1-KO IPEC-J2 + AFB1. Each group consisted of three independent biological replicates (*n* = 3).

Total RNA was extracted according to standard procedures, and RNA concentration, purity, and integrity were evaluated using an Agilent 2100 Bioanalyzer (Agilent, Santa Clara, CA, USA). Only RNA samples meeting the quality requirements for sequencing were used for library construction. RNA sequencing libraries were prepared according to the manufacturer’s standard instructions and sequenced on an Illumina NovaSeq 6000 platform (Illumina, Inc., San Diego, CA, USA) using a paired-end sequencing strategy. Raw sequencing reads were processed to remove adaptor sequences and low-quality reads, generating clean reads for downstream analyses. Clean reads were aligned to the *Sus scrofa* reference genome (Sscrofa11.1) using HISAT2 (v2.2.1), and gene expression levels were quantified using featureCounts (v2.0.8) [31,32]. Differential expression analysis was performed using DESeq2 (v1.50.2) [33]. Genes with an adjusted *p* value < 0.05 and an absolute log2 fold change (|log2FoldChange|) ≥ 1 were considered significantly differentially expressed. Functional enrichment analyses of differentially expressed genes were conducted based on Gene Ontology (GO) terms and Kyoto Encyclopedia of Genes and Genomes (KEGG) pathways using the Metascape online platform (https://metascape.org/gp/index.html, accessed on 17 April 2026). The raw sequencing data have been deposited in the Genome Sequence Archive of the National Genomics Data Center under accession number CRA036578, which are publicly accessible at https://ngdc.cncb.ac.cn/gsa (accessed on 17 April 2026) [34,35].

### 4.14. CGNL1-Dependent AFB1-Responsive Genes Identification

To identify *CGNL1*-dependent AFB1-responsive genes, two contrasts were analyzed: EV IPEC-J2 cells + AFB1 vs. EV IPEC-J2 cells and *CGNL1*-KO IPEC-J2 + AFB1 vs. CGNL1-KO IPEC-J2. Genes with an adjusted *p* value < 0.05 and |log2 fold change| ≥ 1 were considered significantly differentially expressed. For each gene, log2 fold changes from both contrasts were plotted in a scatter plot to visualize consistency and divergence between conditions. CGNL1-dependent AFB1-responsive genes were defined as genes showing significant differential expression in EV IPEC-J2 cells + AFB1 vs. EV IPEC-J2 cells (|log2FC| ≥ 1 and padj ≤ 0.05), but exhibiting minimal change in CGNL1-KO IPEC-J2 + AFB1 vs. CGNL1-KO IPEC-J2 (|log2FC| ≤ 0.25). Functional enrichment (GO/KEGG) of CGNL1-dependent AFB1-responsive genes was performed by the Metascape online platform.

Protein–protein interaction (PPI) network analysis was performed for the CGNL1-dependent AFB1-responsive genes set using the STRING database (https://string-db.org, accessed on 17 April 2026) [36]. *CGNL1*-dependent AFB1-responsive genes were uploaded to STRING. A minimum interaction confidence threshold was applied with the required score set to 0.400 (medium confidence). MCODE (version 2.0.2) was used to identify the most significant modules in a PPI network. Applying Cytoscape (v.3.10.4) (https://cytoscape.org, accessed on 17 April 2026) software for the analysis of the PPI network [37]. Hub genes were identified based on MCC topological analysis in cytoHubba (v0.1) [38].

### 4.15. Statistical Analysis

Data are presented as the mean ± standard deviation (SD). Statistical analyses were carried out using R software (v4.5.2). One-way analysis of variance (ANOVA) was used to evaluate differences among multiple groups, and *p* < 0.05 was considered statistically significant.

## 5. Conclusions

In summary, this study demonstrates that AFB1 induces marked cytotoxicity in porcine intestinal epithelial IPEC-J2 cells, characterized by reduced cell viability, suppressed antioxidant capacity, excessive ROS accumulation, and broad inflammatory and stress-related transcriptomic remodeling. CGNL1 knockout significantly alleviated these effects, preserving redox homeostasis, reducing ROS generation, and attenuating AFB1-induced transcriptional perturbations. CGNL1-dependent AFB1-responsive genes and pathway analyses further suggest that the protective phenotype of CGNL1 deficiency is associated with suppression of the immune response-related gene module. Overall, our findings identify CGNL1 as a potential modulator of epithelial susceptibility to AFB1. It provides new insights that targeting junction-associated signaling components may be a potential strategy to improve resistance to mycotoxin-induced intestinal injury.

## Data Availability

The raw sequencing data have been deposited in the Genome Sequence Archive of the National Genomics Data Center under accession number CRA036578, which are publicly accessible at https://ngdc.cncb.ac.cn/gsa, accessed on 17 April 2026.

## Supplementary Materials

The following supporting information can be downloaded at: https://www.mdpi.com/article/10.3390/ijms27093928/s1.

## Abbreviations

The following abbreviations are used in this manuscript:

AFB1	Aflatoxin B1
IPEC-J2	intestinal porcine epithelial cell line
CGNL1	Cingulin-like 1
WT	Wild-Type
KO	Knockout
EV	Empty vector
FC	Fold Change
CCK-8	Cell counting kit 8
TPM	Transcripts Per Million
DEGs	Differentially Expressed Genes
GO	Gene Ontology
KEGG	Kyoto Encyclopedia of Genes and Genomes
