# Supplementary material for: CRISPR/Cas9-Mediated Knockout of CGNL1 Confers Resistance to Aflatoxin B1 in Porcine Intestinal Epithelial Cells via Suppressing ROS Generation
Source: Int J Mol Sci. 2026 Apr 28;27(9):3928. doi: 10.3390/ijms27093928 (PMC13163822; doi:10.3390/ijms27093928)

Supplementary Data S1. Sanger sequencing of the CGNL1 target locus in IPEC-J2 cells.

The boxed region indicates the sgRNA target

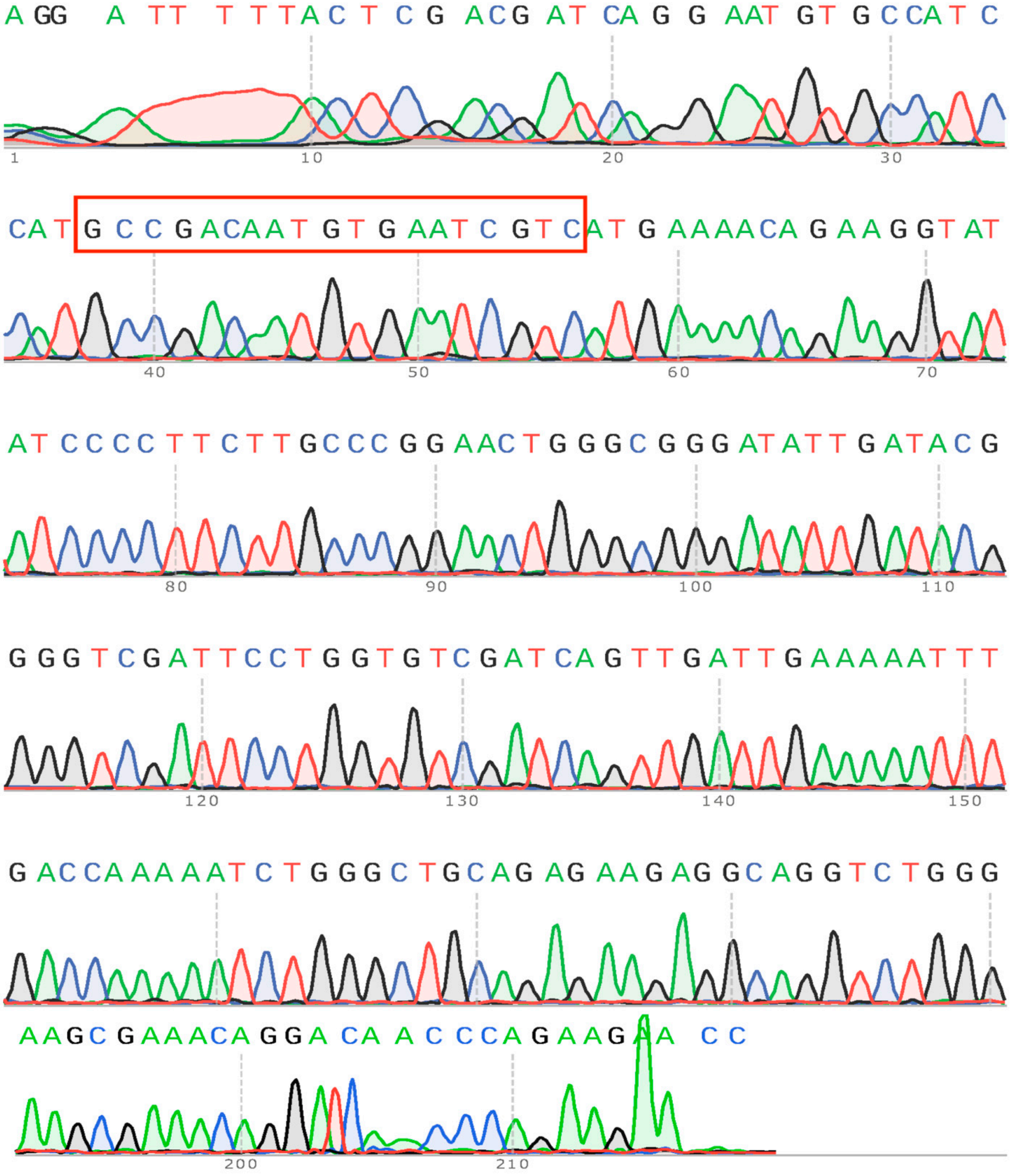

Supplementary Data S2. Sanger sequencing of the CGNL1 target locus in CGNL1-knockout IPEC-J2 cells.

The boxed region indicates the edited site showing indel-associated sequence alteration in CGNL1-KO cells

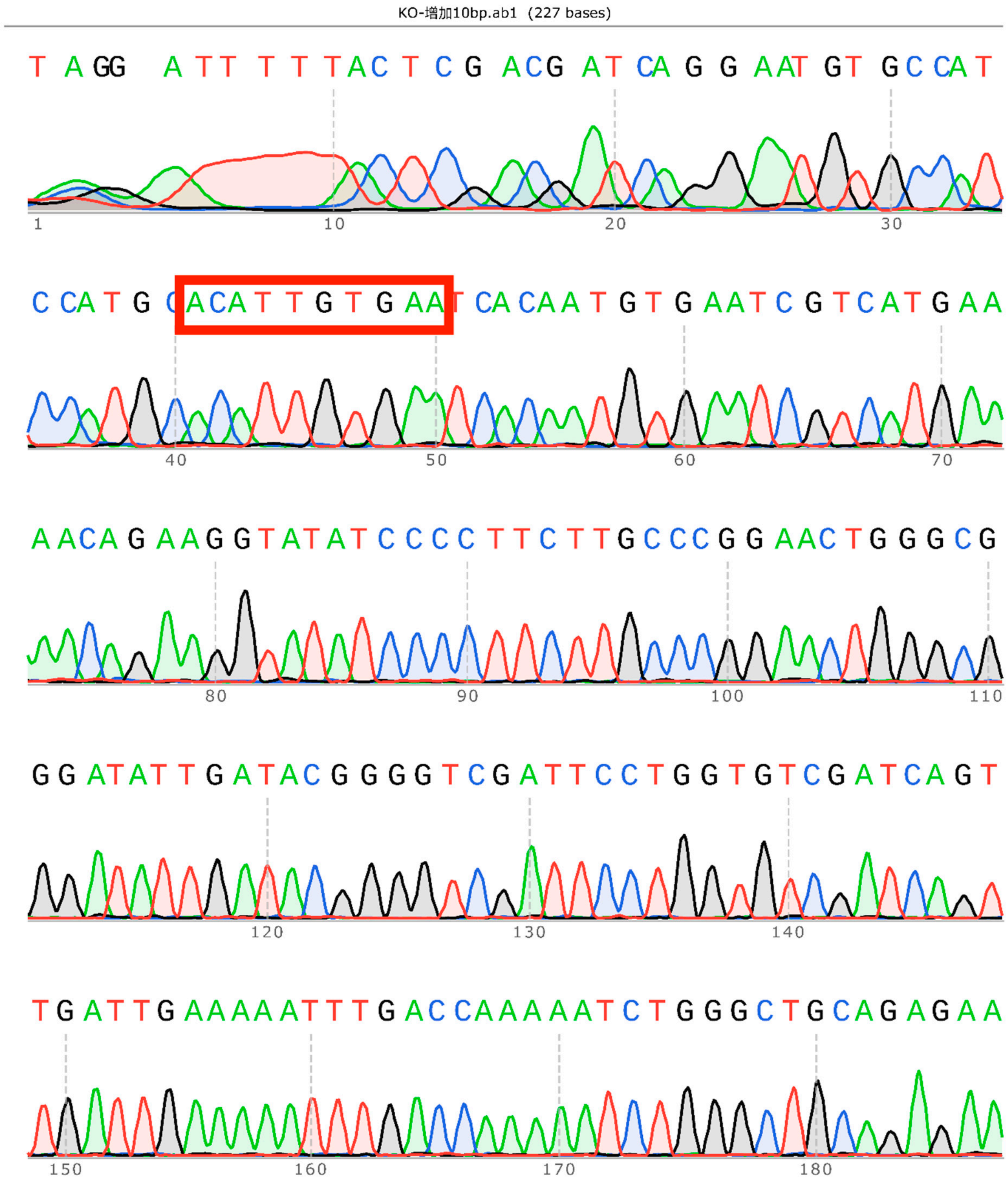

Supplement: Supplementary file 1 [file ijms-27-03928-s001.zip › supplementary data of figures.pdf]
